# miRNA interventions serve as ‘magic bullets’ in the reversal of glioblastoma hallmarks

**DOI:** 10.18632/oncotarget.5926

**Published:** 2015-09-30

**Authors:** Luyue Chen, Chunsheng Kang

**Affiliations:** ^1^ Laboratory of Neuro-Oncology, Department of Neurosurgery, Tianjin Neurological Institute, Tianjin Medical University General Hospital, Tianjin, China

**Keywords:** glioblastoma, microRNAs, miRNA-targeting strategies

## Abstract

microRNAs (miRNAs) are no longer deemed small pieces of RNA “trash” in the human transcriptome but are considered to be master regulators of gene expression that are critical in maintaining cellular homeostasis post-transcriptionally. The concept triggers great interest in studying miRNA dysregulations in human diseases, especially in cancers. Glioblastoma (GBM) has long been the leading cause of the high mortality and morbidity of CNS tumors in adults, which is a consequence of the lack of strategies to reverse the hallmark features of GBM (e.g., borderless expansion and diffuse infiltration). In the past decade, dissecting the molecular architecture of GBM has led to a better understanding of the molecular basis of the hallmarks, generating many promising pharmacological protein targets. However, few clinical responses have been highlighted, suggesting the demand for new therapeutic strategies and targets. In this review, we systemically summarize the context-dependently validated miRNAs with one or more functional targets in the development of GBM hallmarks and review the current miRNA-targeting strategies. We note that only a few miRNA-based therapeutics are trialed for clinical significance, and none of them is tailored to GBM, thereby urging us to bring miRNA therapeutics to the front line either alone or in combination.

## INTRODUCTION

Glioblastoma (GBM), the WHO grade IV tumor of the CNS, is the most prevalent and aggressive glioma variant in adults [1]. Gross total surgical excision followed by involved-field radiotherapy up to a total dose of 60 Gy was the only accepted GBM management by physicians for decades, as no chemotherapeutic agents significantly improved the survival of GBM patients until the introduction of temozolomide, an oral alkylating agent. However, the median survival of 14 months, 27% of the 2-year and 10% of the 5-year progression-free survival rates still remain dismal for both patients and physicians [2]. More efforts need to be made to change the poor prognosis of GBM patients.

Over a decade, the interplay between the protein-coding and non-protein-coding genome, especially the miRNAome (microRNA genome), has been the most exciting yet unexpected discoveries in oncology. microRNAs (miRNAs) are approximately 22 nt-long small non-coding RNA molecules (ncRNAs) that regulate gene expression at the post-transcriptional level. Most miRNAs can be transcribed from the intergenic or intronic regions by RNA polymerase II. Then the primary transcripts (pri-miRNAs) from intergenic regions undergo nuclear processing into a stem-loop precursor of approximately 70 nt (pre-miRNA) by the Drosha-DGCR8 (DiGeorge syndrome chromosome region 8) complex, a nuclear RNase III complex, while the intronic transcripts bypass Drosha processing [3]. Subsequently, exportin-5 mediates the nuclear export of the correctly processed pre-miRNA in a Ran guanosine triphosphate (RanGTP)-dependent manner. Following the export of pre-miRNA from the nucleus, Dicer, a cytoplasmic RNase III that commonly forms a complex with TAR RNA binding protein (TRBP), binds to the pre-miRNA and further processes the hairpin miRNA precursors into approximately 22 nt miRNA duplexes [4]. The duplexes are unstable and soon cleaved into single-stranded mature miRNAs. Mature miRNAs are then incorporated into the miRNA induced silencing complexes (miRISCs) to exert their post-transcriptional function by seed region (5′ region of the miRNA centered on nucleotides 2-7) complementarity [5]. The function of mature miRNAs has been widely studied and yet hotly debated, and is predominantly considered to mediate the silence of mRNA translation [6]. Due to great scientific interest worldwide, the amount of miRNAs in various species has grown exponentially and now includes 1881 miRNA precursors and 2588 mature sequences that have been documented in humans by miRBase (Release 21, June 2014, http://mirbase.org/cgi-bin/browse.pl?org=hsa). It is estimated that over 60% of human protein-coding genes are conserved targets of miRNAs, unveiling previously unnoticed, extensive RNA-RNA interacting networks in cellular homeostasis maintenance [7]. In the networks, miRNAs are commonly regarded as active regulators, whereas their target mRNAs are the passive receptors of repression, and thus how mRNAs affect miRNA functions is less well characterized. Recently a hypothesis confers a non-protein-coding function on those protein-coding mRNAs, given that mRNAs may influence each others’ levels by competing for a limited pool of shared miRNAs, thus acting as competing endogenous RNAs (ceRNAs) [8–10]. Moreover, as our knowledge of the transcriptome space has expanded, several other RNA species, including large intergenic non-coding RNAs (lincRNAs) [11–13], transcribed ultraconseverved regions (T-UCRs) [14], pseudogenes [15–17] and circular RNAs (circRNAs) [18, 19], may also serve as ceRNAs, functioning *in trans* to regulate levels of free miRNAs and consequently other RNAs. However, compared with the *cis* regulatory networks, ceRNA research is obviously in its infancy, and more compelling evidence is still required to ascertain whether ceRNA crosstalk represents a widespread network of RNA regulation.

As both the guide and passenger strand-derived miRNAs are biologically functional, in this review, we prefer to use the latest nomenclature proposed by the miRBase to substitute for the previously assigned names for miRNAs [20]. For example, with respect to miR-21, miR-21 and miR-21* were previously assigned to nominate the guide and passenger strand processed from the miR-21 stem-loop precursor, respectively. The new nomenclature is started to assign names of the form miR-21-5p and miR-21-3p for the individual sequences derived from the 5′ and 3′ arm of the miR-21 stem-loop precursor [21]. As a hallmark of the better understanding of the nature of miRNA species, the new nomenclature will be used in this review to denote the new miRNA annotations (see Supplementary Data for the previous IDs of the miRNAs mentioned in this review).

In 2005, the first miRNA dysregulation was identified in GBM [22]. miR-21-5p detected by northern blot was overexpressed in GBM tissues when compared with non-neoplastic human control tissues. Moreover, the suppression of miR-21-5p function led to decreased cell number and increased apoptosis and caspases activation, suggesting that aberrantly expressed miR-21-5p is an antiapoptotic factor in GBM [22]. Meanwhile, a systemic screen for miRNA aberrations by microarray of 245 miRNAs in GBM samples first identified a set of dysregulated miRNAs, including the upregulation of miR-10b-5p, miR-21-5p and miR-25-3p, and downregulation of miR-128-3p and miR-181a-5p/181b-5p/181c-5p [23]. Since then, it has been documented that miRNA dysregulation could play important roles in GBM development and progression.

## MIRNAS IN HALLMARKS OF GBM

**Figure 1:**
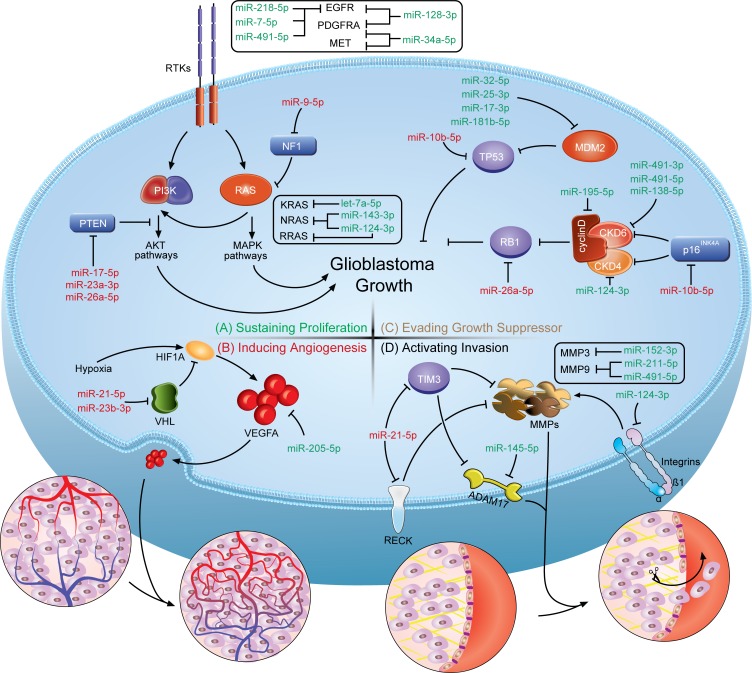
A schematic illustration of how miRNAs involve in the genesis of GBM hallmarks The figure shows the crosstalks of miRNAs and protein-coding genes identified in multiple nodes of the pathways on developing GBM hallmark features. (Top left, **A.**) Sustaining proliferation can be triggered by alterations within the receptor tyrosine kinase (RTK) signaling circuits. (Bottom left, **B.**) Inducing angiogenesis in GBM relies greatly on the expression of hypoxia-induced VEGF family proteins. (Top right, **C.**) Evading the prototypical tumor suppressors, RB1 and TP53, enables tumor cells to circumvent growth control. (Bottom right, **D.**) Activating invasion requires degradation of ECM components by metalloproteinases to overcome the dense matrix. The miRNA-mRNA crosstalks turn the short ‘nonsense’ sequences into endogenous oncogenes or tumor suppressors. Based on their distinct functions, oncomiRs are colored in red and tumor suppressive miRNAs are in green.

Incurable GBM is characterized by uncontrolled cellular proliferation, robust angiogenesis, intense resistance to apoptosis, diffuse infiltration, a propensity for necrosis and rampant genomic instability [24]. The hallmark features are preserved, at least partially, by biological capabilities of sustaining proliferative signaling, inducing angiogenesis, evading growth suppressors and activating invasion and metastasis. The current understanding of these GBM hallmarks has already gone beyond the protein-coding genes and focused increasingly on the non-coding genome, especially the miRNAome. It is generally accepted that hallmark features of GBM are not only the reflection of chaos in protein function of certain pathways but also the consequence of the dysregulation of miRNA-mediated translation control (one of the typical examples is the initiation of The Cancer Genome Atlas (TCGA) Pan-Cancer analysis project) [9, 25–27]. The miRNA-mRNA interactions turn the short ‘nonsense’ sequences into endogenous oncogenes or tumor suppressors. Moreover, deregulation of miRNAs in cancers is unlikely to be a random event, but instead they have certain expression patterns. miRNA expression patterns could define a tumor type, implying that certain changes in miRNAs might drive the malignant transformation to a particular cancer [27–30]. Therefore, whether a miRNA acts as an oncogene (oncomiR) or tumor suppressor depends on the regulated genes and cellular context. In this section, we only discuss the miRNAs that have experimentally validated targets in GBM (Figure 1).

**Sustaining proliferative signaling**. Uncontrolled cell proliferation in GBM could be triggered by excessive proliferative signals caused by somatic alterations within the receptor tyrosine kinase (RTK) signaling circuits. The activation of intracellular kinase domains of RTKs results in the activation of a complex network of intracellular signaling cascades, the most studied of which are the phosphatidylinositide 3-kinase (PI3K)/AKT and RAS/mitogen-activated protein kinase (MAPK) pathways (Figure 1A) [31]. Abnormally augmented signals downstream from RTKs enable cancer cells to sustain proliferation that is under tight control in normal cells. Therefore, miRNAs, serving as the important post-transcriptional regulators, would possibly account for attenuating or deteriorating the molecular dysregulation caused by the genomic alterations. The crosstalks between miRNAs and RTK signaling circuits enroll miRNAs in the modulation of proliferative features of GBM. At least 90% of GBM cases harbor genetic alterations in RTK pathways. *Epidermal growth factor receptor (EGFR), platelet-derived growth factor receptor alpha polypeptide (PDGFRA), MET proto-oncogene (MET)* and *fibroblast growth factor receptor (FGFR)* are among the most commonly dysregulated RTKs in GBM [27, 32]. However, no miRNA dysregulations correlated with the deregulation of FGFR in GBM has been reported yet. EGFR and PDGFRA are well-established oncogenes in GBM [24], and thus the identification of their miRNA regulators following the discovery of functional implications of miRNAs in cancers is warranted. Moreover, the nature of miRNA target recognition defines the miRNA-mRNA interactions as the many-to-many relationship. miR-7-5p [33], miR-128-3p [34], miR-491-5p [35] and miR-218-5p [36] coordinately regulate the expression of EGFR in human GBM. As the negative regulators of EGFR expression, these miRNAs were shown to be downregulated in GBM tissues and/or cell lines to drive gliomagenesis. The restoration of EGFR-targeting miRNA expression significantly decreased EGFR protein synthesis and as a result, impaired the proliferative capability of GBM cells [33–36]. Apart from direct repression of EGFR expression, miR-128-3p was shown to simultaneously target PDGFRA [34]. Additionally, PDGFRA is also a direct target of miR-34a-5p, a downregulated miRNA in the proneural subtype compared with the mesenchymal subtype of GBM [37]. It is of particular interest that the proneural subtype is characterized by *PDGFRA* genetic alterations in conjunction with exclusively high levels of PDGFRA expression and irresponsiveness to aggressive treatments [38]. Derepression of PDGFRA by the downregulation of miR-34a-5p in the proneural subtype that results in a more proliferative phenotype may be responsible for the difference in response to clinical treatment for GBM. Gene mutation or amplification in *MET* (1.6%), when compared to *EGFR* (57.4%) or *PDGFRA* (13.1%), is a relatively rare event in GBM [27]. But as a mesenchymal marker, MET is highly expressed in the mesenchymal subtype of GBM [38]. It is interesting that shared miRNA of MET and PDGFRA, miR-34a-5p, is also highly expressed in the mesenchymal subtype [37, 39]. The same tendency of miR-34a-5p and MET expression in the mesenchymal subtype suggests an underlying regulatory network that remains to be further elucidated.

Downstream effectors of receptor tyrosine kinases, PI3K and RAS, are less studied targets of miRNAs involved in gliomagenesis. However, TCGA reported that PI3K and RAS activation might be the obligatory events in developing GBM [27, 32]. In GBM, no miRNAs has been validated yet to target the PI3K p110 and/or p85 subunits that are responsible for the activation of downstream AKT signaling proteins [40]. In contrast, the miRNA regulators of RAS are better characterized. The biological functions of the RAS family (Harvey rat sarcoma viral oncogene homolog (HRAS), Kirsten rat sarcoma viral oncogene homolog (KRAS) and neuroblastoma RAS viral oncogene homolog (NRAS)) have been extensively studied for decades. In response to activated growth factor receptors, RAS activation affects downstream effectors, including MAPK and PI3K [41]. Though only 1% of the GBM tumors have a *RAS* mutation or amplification, 10% of GBM tumors contain *neurofibromin 1* (*NF1)* inactivating genetic alterations that lead to hyperactive RAS activity by enhancing the intrinsic GTPase activity [27, 42]. Three recent reports focused on miRNAs targeting RAS in GBM and showed that miR-143-3p directly targets NRAS [43], let-7a-5p directly targets KRAS [44], and both NRAS and RRAS (related RAS viral oncogene homolog, HRAS homolog) are direct targets of miR-124-3p [45]. Moreover, these miRNAs are all downregulated in GBM samples and cell lines, underlying the malignant transformation [43–45].

Excessive proliferative signaling from another point of view is the consequence of a disruption of negative-feedback mechanisms at multiple nodes within the proliferative circuitry. Phosphatase and tensin homolog (PTEN) and NF1, which counteract PI3K and RAS, respectively, are among the most important negative regulators of the proliferative pathways. The inactivated genetic mutations of *NF1* and *PTEN* are found in 10% and 41% of GBM cases, respectively [27]. With respect to NF1, it is targeted by miR-9-5p, a miRNA upregulated in GBM that adds to the complexity of NF1 and downstream RAS signaling deregulation [46, 47]. Similarly, the negative regulation of PI3K signaling by PTEN is crucial for proper proliferative signal transduction [48]. Three upregulated miRNAs, miR-23a-3p, miR-26a-5p and miR-17-5p, directly target PTEN in GBM [47, 49–51]. As a consequence, the upregulation of PTEN-targeting miRNAs provides a bypass to activate PI3K pathways. In recent years, targeting RTK pathways has been one of the most exciting developments in cancer research, and some of the targets have been clinically validated. But in GBM, the utilization of such molecularly targeted drugs only received modest clinical benefits. By the understanding of the importance of miRNAs, miRNA-based treatments should also be taken into consideration either alone or in combination.

**Inducing angiogenesis**. Malignant tumors addictively rely on the formation of new blood vessels. It is of particular importance in brain tumors that glomeruloid microvascular proliferation is a hallmark of GBM and is one of the diagnostic criteria applied to distinguish the high-grade gliomas from the low-grades [52]. Therefore, inhibiting angiogenesis has long been regarded as a promising strategy in GBM. These tumors stimulate new blood vessel formation through processes driven primarily by vascular endothelial growth factor A (VEGFA), the most established proangiogenic protein in the VEGF family (Figure 1B). The overexpression of VEGFA and subsequent activation of its receptors is an important event during glioma progression [53]. miR-205-5p, a miRNA significantly downregulated in GBM, was found to directly interact with the VEGFA 3′-UTR and decreased VEGFA expression in GBM cell lines [54]. The downregulation of miR-205-5p could be, at least partially, responsible for the overexpression of VEGFA in GBM. Additionally, VEGFA upregulation can be induced by hypoxia, a condition that commonly occurs in GBM due to the highly proliferative nature [55]. This adaptive change relies considerably on the increase in the stability of hypoxia inducible factor 1 alpha subunit (HIF1A), which is negatively regulated by the von Hippel-Lindau (VHL) tumor suppressor [56]. Of note, miR-21-5p and miR-23b-3p are reported to target VHL and decrease the production of the VHL protein, upregulating VEGFA expression [57, 58]. Moreover, miR-7-5p downregulates the expression of O-linked N-acetylglucosamine transferase (OGT), leading to decreased expression of vascular endothelial growth factor receptor 2 (VEGFR2) [59]. Attempts to target angiogenesis using the VEGF antibody was previously considered to be promising in GBM. However, recently, the disappointing results from the VEGF-targeting agents in treating GBMs diminishes the enthusiasm for such approaches [60], suggesting that new therapeutic targets or strategies need to be developed. To make progress, better understanding of the miRNAs contributing to angiogenesis in high-grade gliomas will lead to a better understanding of the highly vascularized nature of the tumors and more effective treatments can be developed.

**Evading growth suppression**. Apart from the ability of inducing and sustaining growth-stimulatory signals, GBM cells must also circumvent biologically programmed pathways that negatively regulate cell proliferation. Many tumor suppressive genes have been established through gain- or loss-of-function experiments in cell lines or animal models. Among those well-established suppressors, there are two prototypes, retinoblastoma 1 (RB1) and tumor protein p53 (TP53) (Figure 1C). Similarly, better understanding the dysfunction of the RB1 and TP53 pathway should also implicate the roles of dysregulated miRNAs. With regards to *TP53*, the guardian of the genome in response to various stress signals, it is frequently mutated or deleted in 28% of GBMs [27]. The loss-of-function mutation of TP53 results in the inability to stop further cell-cycle progression triggered by oncogenic signals [61]. miR-10b-5p, one of the most studied miRNAs in GBMs, is highly upregulated in human GBM and pleiotropically regulates invasion, angiogenicity and apoptosis of GBM cells resembling the mesenchymal subtype. The pleiotropic effects of miR-10b-5p is due to its suppression of multiple tumor suppressive genes, including TP53 [23, 62, 63]. miR-10b-5p directly targets TP53 in GBM, giving the tumors a way to evade growth control and enable persistent cell proliferation by perturbing the miRNAs expression. Meanwhile, TP53 signaling is under the precise control of the negative regulator, mouse double minute 2 (MDM2). MDM2 regulates the ubiquitin-dependent degradation and transcriptional activity of TP53 [64]. MDM2 mRNA is upregulated in both GBM cell lines and samples [65], and the upregulation could be a consequence of the downregulation of miR-17-3p, miR-181b-5p, miR-25-3p or miR-32-5p, which directly target MDM2 gene expression [50, 65, 66]. It is worth noting that miR-25-3p and miR-32-5p are two miRNAs repressed by TP53, suggesting a feedback circuit between TP53 and MDM2 mediated by miRNAs [65]. The feedback circuit can explain the overexpression of miR-25-3p in GBM reported by several separate studies, in which the miRNA is meant to be downregulated to increase MDM2 expression and thus inactivate TP53 [23, 47, 67]. In addition, miR-181b-5p is downregulated in GBM samples, further indicating that miRNA compromises contribute to the complexity of the pathological progression of gliomas [66].

Another key tumor suppressive pathway is the RB1 pathway. The p16^INK4a^-cyclin-dependent kinase (CDK) 4/6-RB1 axis is responsible for the tight regulation of RB1 activity in both cycling and non-cycling cells [68]. Approximately 80% of GBMs have one or more alterations affecting the RB1 function, including direct *RB1* mutation/deletion, *CDK4/6* amplification and *cyclin-dependent kinase inhibitor 2A (CDKN2A)* deletion (encoding both p16^INK4a^ and p14^ARF^) [27, 32]. Due to the frequent alterations in GBM, the majority of our current functional investigations are focused on those mutated genes and the contribution to gliomagenesis. miRNA functional studies, which mostly rely on the established gene functions, are also performed. miR-26a-5p, which has been discussed previously as targeting PTEN, also targets RB1 in GBM. Interestingly, in U87 cell lines lacking the expression of PTEN, miR-26a-5p was still capable of inhibiting tumor growth, and the overexpression of PTEN or RB1 could both antagonize the proliferative effects of miR-26a-5p [69]. Besides, CDK-cyclin complexes mediated phosphorylation is one of the main mechanisms of inactivation of RB1 protein [70]. The frequent gain-of-function mutations on *CDK4/6-cyclin D* complexes underscore their importance and potential in the development and progression of GBM. Therefore, the regulation of key components of this RB1 regulatory complex provides rationales for RB1 functional normalization. miR-124-3p is reported to radiosensitize human glioma cells by downregulating CDK4 [71], while CDK6 is a direct target of miR-138-5p and miR-491-3p/5p [35, 72]. miR-195-5p is the miRNA regulator of cyclin D1, a cyclin partner of CDK4/6 [73]. Notably, those miRNAs that target CDK4/6-cyclin D1 complexes are all downregulated in GBM samples [35, 67]. *CDKN2A* deletion is one of the most frequent mutations in GBM, resulting in the loss of two tumor suppressors (p16^INK4a^ and p14^ARF^). p16^INK4a^ can bind specifically to CDK4/6 and inhibit the catalytic activity of the CDK4/6-cyclin D complexes [74]. miR-10b-5p, in addition to targeting TP53 in GBM, also targets p16^INK4a^, and the inhibition of miR-10b-5p leads to cell cycle arrest [63]. Collectively, dysregulated miRNAs could be an underlying avenue for GBM to elude the tumor suppressor and play far more important roles in gliomagenesis than previously thought.

**Activating invasion and metastasis**. The propensity of glioma cells to move and invade the brain, which enables the tumor to elude whole surgical resection and chemoradiation therapy, remains the major obstacle to improve the poor outcomes of GBM patients. Though GBM is a type of highly infiltrative cancer, it is estimated that less than 2% of GBM cases metastasize outside the CNS, which distinctly differs from other solid tumors [75]. Due to the exclusive nature of metastasis, GBM cells may prefer to migrate through the tortuous extracellular spaces of the brain rather than the vascular or lymphatic duct system like many other solid tumors do. It is, thus, supposed that the interaction of invading glioma cells with the extracellular matrix (ECM) is crucial in the initiation of invasion and migration. Generally, cell attachment is mediated by interactions between cell-cell and cell-ECM receptors, including integrins, cadherins and neural cell adhersion molecules, and degradation of ECM components by metalloproteinases is essential for cell detachment [76]. With the understanding of the molecular basis of invasion, several miRNAs are associated with the invasiveness of GBM cells (Figure 1D). The matrix metalloproteinases (MMPs) and a disintegrin and metalloproteinases (ADAMs) are two distinct types of secreted metalloproteinases expressed by migrating glioma cells to overcome the dense matrix that fills the extracellular space, and the proteolytic activity can be blocked by endogenous metalloproteinase inhibitors, such as tissue inhibitor of metalloproteinase (TIMPs) and reversion-inducing-cysteine-rich protein with kazal motifs (RECK) [77]. MMP9 (a gelatinase that binds and degrades gelatin) is a direct target of miR-491-5p and miR-211-5p, which are both upregulated in tumor samples [35, 78, 79], and MMP3 (an archetypal metalloproteinase with stromelysin activity) is targeted by miR-152-3p [80]. Additionally, ADAM17, a non-MMP, is under the direct regulation of miR-145-5p [81]. It is obvious that if glioma cells lose the control of MMPs and ADAMs by miRNAs, the ECM homeostasis cracks and the combined activity of these proteases remodels the ECM to favor tumor invasion. miR-21-5p, in addition to regulating metalloproteinases directly, targets TIMP3 and RECK, two negative metalloproteinase regulators [82]. Notably, miR-21-5p is shown to be markedly elevated in GBM tissues by many separate studies [22, 23, 47, 67]. As the MMPs and ADAMs each comprise more than 20 members, targeting a single target seems to be inessential in cancerous diseases, which provides miR-21-5p with an opportunity to broadly inhibit metalloproteinase function. Besides, interactions with the ECM are mostly mediated by integrins, which enable cells to sense the extracellular environment and adjust their behavior to environmental cues [83]. A previous study demonstrated that treating GBM cells with an anti-β1 integrin antibody significantly reversed the increased adhesion, invasion and MMP2 activity induced by irradiation [84]. The overexpression of β1 integrin was observed in GBM patients and might be a result of the loss of its miRNA regulator. miR-124-3p, frequently downregulated in GBM, targets β1 integrin and is shown to affect glioma cell migration and invasion *in vitro* [85]. In sum, the roles of miRNAs in glioma cell invasion or migration further our understanding on the genesis of the aggressive glioma phenotype. Some of them have been discovered, whereas more still remain to be found. The blockade of the excessive pro-invasive miRNAs or restoration of the lessened anti-invasive miRNAs will provide extra treatment options for advanced-stage gliomas that are marked with poor prognoses for decades.

## STRATEGIES IN MOLECULARLY TARGETING MIRNAS

As miRNAs are attracting more and more attention due to their functionalities in cancer, the normalization of miRNA expression is expected to shed light on their therapeutic potential. The molecular basis of targeting such small RNA molecules rests upon our understanding of both their biogenesis processes and functioning mechanisms. The purposes in the miRNA-based therapeutics are to neutralize the oncomiRs and replenish the tumor suppressive miRNAs. To serve a therapeutic purpose, many specific targeting approaches have been invented and put into practice. The approaches are seemingly diversified and can be cataloged into three main categories: oligonucleotides, expression cassettes and small-molecule drugs.

**Figure 2:**
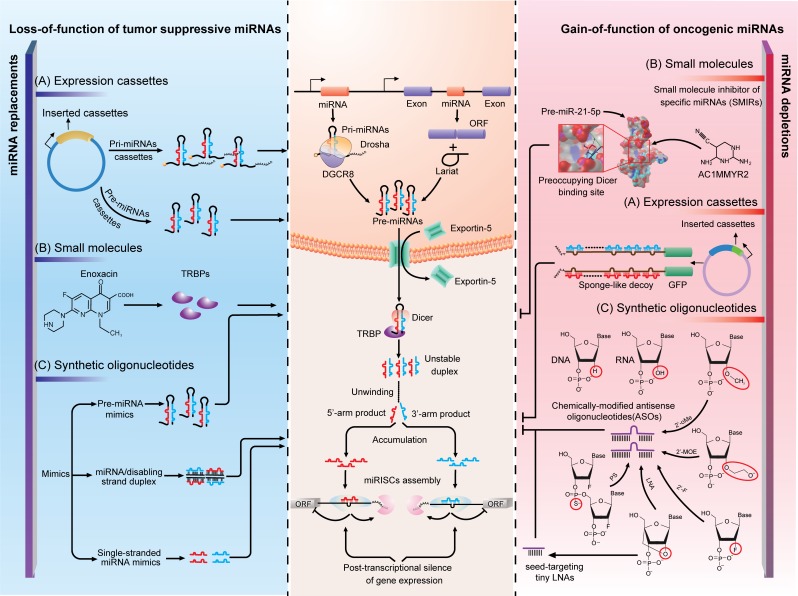
Strategies of miRNA interventions **A.** Expression cassettes-based strategy is the utilization of vectors that ectopically express miRNA or its complement to rectify abnormality in miRNA expression. **B.** Small molecules are less studied but promising drugs for molecularly targeting miRNAs. With distinct mechanisms, enoxacin rescued globally downregulated miRNAs by binding to TRBP to promote miRNAs processing and AC1MMYR2 specifically block Dicer processing of pre-miR-21 to mature miR-21-5p. **C.** Synthetic oligonucleotides are designed to chemically synthesize miRNA mimics or antisense oligonucleotides (ASOs). By using different chemical modifications, ASOs can now bind to miRNAs with high affinity and specificity. In this figure, the left and right panel illustrate the mechanisms of the application of the three strategies in miRNA normalization. The middle panel is the current understanding of miRNA biogenesis and functioning mechanism, which is the molecular basis of miRNA interventions.

**Modified oligonucleotides**. In the past decade, one of the most inspiring discoveries in oncology and oncology pharmacology has been to establish the functional roles of miRNAs, particularly their roles in human malignancies. miRNAs can act context-dependently as tumor suppressors or oncogenes, which provides the rationales for targeting the oncomiRs by antisense oligonucleotides (ASOs) and the tumor suppressive miRNAs by miRNA mimics (Figure 2C).

The inactivation of an upregulated or overexpressed miRNA is important for the loss-of-function study and should be a prime approach in future cancer management. However, at first, the enthusiasm was curbed by the lack of effective approaches to inhibit miRNA expression. Traditional genetic approaches are difficult as miRNAs are unexpectedly small in size, sporadically located within introns of protein-coding genes and frequently co-transcribed in clusters. It is the inhibitory use of unmodified antisense DNA oligonucleotides against miR-2a-3p and miR-13a-3p in Drosophila melanogaster that first overcame these [86]. Antisense oligonucleotides function through competitive base-pairing to mature miRNA and inducing the degradation or stoichiometric duplex formation. However, the susceptivity of the unmodified DNA oligonucleotides to endogenous nuclease mediated degradation and a high working concentration (μM) limited the *in vivo* utility, which, meanwhile, sparked interest in improving the stability and affinity of this type of antisense molecule [87]. Before long, two research groups demonstrated the ability to overcome the defects by replacing single-stranded DNAs (ssDNAs) with ssRNAs, as DNA has a lower affinity for the single-stranded RNA/miRNA than RNA does for itself, and the addition of the 2′-O-methyal (2′-OMe) modification increased ribonuclease resistance and maintained or improved affinity and specificity to the target RNA/miRNA [87, 88]. The synthetic single-stranded antisense RNAs with 2′-OMe modifications were capable of reducing the working concentration to a nanomolar scale and reaching satisfactory suppression on siRNA and miRNA induced silencing [87, 88]. Ever since, chemical modifications are considered vital for the design of miRNA-inhibitory oligonucleotides. In addition to 2′-OMe modification, several other modifications at the 2′-position of the sugar ring block target miRNAs effectively, including 2′-O-methyoxyethyl (2′-MOE) [89], 2′-fluoro (2′-F) [90] and locked nucleic acid (LNA) modifications [22]. By comparison, 2′-MOE-modified antisense oligonucleotides displayed better miRNA-inhibitory efficacy than 2′-OMe-modified oligonucleotides, while 2′-F-modified oligonucleotides outperformed both of them [89, 90]. LNA, which is defined as an oligonucleotide that contains one or more 2′-*O*,4′-*C*-methylene-*β*-D-ribofuranosyl monomer(s), is a novel class of nucleic acid analogues. By blocking the RNAs with a methylene bridge, LNA-containing oligonucleotides offer a dramatic increase in binding affinity towards complementary ssRNA or ssDNA, and display excellent mismatch discrimination and nuclease resistance [91]. It is in the GBM that the first LNA-modified anti-miR-21-5p that contains eight central LNA nucleotides designed to target miR-21-5p seed showed a moderate increase in inhibitory efficacy when compared with entire 2′-OMe-modified counterparts [22]. Interestingly, due to the high affinity of LNA-modified oligonucleotides, a seed-targeting 8-mer LNA, termed tiny LNA, was designed to target entire miRNA families. Tiny LNA showed negligible off-target effects in mice bearing breast tumors, providing an opportunity to explore the functions of entire miRNA families *in vivo* [92]. In contrast to various 2′-position modifications, phosphorothioate (PS)-containing backbone modification [93] and cholesterol conjugation [94] are two important ancillary tools for the design of antisense drugs. PS-substituted ASOs are proposed even earlier than the discovery of the 2′-OMe-modified antisense RNAs. Due to the lack of successful strategies to improve miRNA-binding affinity of ASOs, the first miRNA-targeting, PS-modified DNA antisense oligonucleotides failed. PS-modification no doubt enhances the *in vivo* stability to nucleolytic hydrolysis but it also decreases the stability of duplexes with miRNAs, which results in the loss of miRNA-inhibitory function *in vitro* [87]. The first conjunction method reported to refine the performance of anti-miRs *in vivo* is 3′-conjuntion with cholesterol [94]. The efficient and selective uptake of these cholesterol- or other lipophilic-conjunctions depends on the interactions with lipoprotein particles, lipoprotein receptors and transmembrane proteins. The interactions will direct lipophilic oligonucleotide delivery into the liver, gut, kidney and steroidogenic organs [95]. To date, a combination of multiple chemical modification strategies further highlights the promise for improving the potency of antisense drugs. One of the most typical examples is the “antagomiR.” To silence endogenous miR-122-5p, the first antagomiR, antagomiR-122-5p, is designed with asymmetric PS-modifications on both the 5′- and 3′-ends, 2′-OMe modifications and a 3′ cholesterol tail [94]. The antagomiRs are well tolerated, and function satisfactorily both *in vitro* and *in vivo*, indicating a more generally accepted complementarity-based method for miRNA silencing [96, 97]. Notably, almost all of the ASOs are now designed to target the mature miRNAs. An alternative approach is to target overexpressed miRNAs in diseases by disrupting their precursor. Such an approach will circumvent the difficulty in targeting a miRNA without off-target effects affecting other family members, and provide significant flexibility for the design and validation of ASOs. Moreover, this hypothesis can extend to using small interfering RNAs (siRNAs) to knockdown miRNA expression if the siRNAs are specifically designed for either pri-miRNAs or pre-miRNAs, as siRNAs also function within the nucleus [98]. It is interesting to find no literature available on such a strategy. However, there is one concern on therapeutic targeting of miRNA precursors. miRNA hairpins commonly produce two lopsidedly abundant miRNA species, and both of them are functionally correlated with RISC components. Intervention on miRNA precursors will disrupt the production of miRNAs from both arms and inevitably generate accessory miRNA disorders. To avoid this, knockdown or overexpression of a miRNA precursor should build on the understanding of the physiological relevance of the two mature miRNA products. In GBM, a typical example is pre-miR-17 that produces two mature miRNAs with dual roles in tumor growth: it suppresses tumor proliferation but protects cells from cytotoxic agent treatment [50]. Due to the complex functional roles, interference of pre-miR-17 may not be a wise choice.

The restoration of miRNA expression, also termed miRNA replacement, relies on the fact that downregulation of particular miRNAs contributes to the genesis or progression of human diseases. To rescue the miRNA expression, miRNA mimics are synthesized and introduced into cells. According to the sequence identity, the mimics can be subgrouped into mature miRNA mimics and pre-miRNA mimics. Generally, the mature miRNA mimics are small, double-stranded RNA molecules that are optimized by chemical modification to force only the desired 5p- or 3p-miRNA into RISC, while single-stranded mimics can also be used with less biological stability [99]. Currently, the methodologies of synthesis and delivery of a miRNA mimic benefit largely from the progress of siRNA developments as siRNAs receive unprecedented interest in post-transcriptional gene silencing. However, compared with siRNAs, miRNA mimics have an incomparable advantage over siRNAs. Synthetic mimics are expected to behave like the depleted, naturally occurring miRNA, and therefore, are manipulated to target multiple oncogenes and pathways. miR-34a-5p mimics, for example, simultaneously suppressed CDK4, MET and B-cell CLL/lymphoma 2 (BCL-2) in lung cancer xenografts by intravenous injection. Moreover, systematic evaluation of miR-34a-5p mimics-treating mice suggested that the treatment is well tolerated and does not induce an immune response [100]. Recently, the chemically synthesized pre-miRNA mimics, RNA hairpins with sequences identical to natural pre-miRNAs, have been proposed due to the increasing evidence concerning the biologically functional 5p- and 3p-miRNAs [101]. Delivered into cells, pre-miRNA mimics undergo further processing by Dicer into functionally active 5p- and 3p-derived mature miRNAs. For example, pre-miR-34a mimics produce two active miRNAs to synergistically regulate tumor necrosis factor (TNF) activity. Reintroduction of pre-miR-34a, rather than either mature product, represents a more ideal therapeutic option [101].

**Expression cassettes**. The expression cassette-mediated miRNA interventions utilize several commercially available vectors, such as plasmids and more commonly, viral vectors (lentiviruses, retroviruses and adenoviruses), to construct vehicles for the expressing cassettes containing miRNA or its complement (Figure 2A). In one attempt, the insertion of pri- or pre-miRNAs sequence resupplies the tumor suppressive miRNAs, aiming at decreasing the oncogene expression and ablating the oncogenic activity. This technique has been conceptually accepted and almost all of the constructs are commercially available. However, the expressing vectors overexpress miRNAs from both arms similar with the introduction of the synthetic pre-miRNAs, and most of the time one of them is undesired. Therefore, minor mutations on the seed regions of the undesired miRNA can be made to avoid side effects. In another attempt, many research groups constructed their own vectors, usually viral vectors, to overexpress cassettes that contain multiple miRNA binding sites to saturate unbounded target miRNAs. To date, the methodology has been further meliorated by different groups with different names, such as sponge [102], decoy [103], eraser [104] and antagomir [105]. The use of viral vectors with strong promoter is to ensure supraphysiological levels of target sequences. Among those sponge-like constructs, the most well studied one is the “miRNA sponge”, which is constructed by inserting tandemly arrayed miRNA binding sites into the 3′-UTR of a reporter gene encoding destabilized green fluorescent protein (GFP) driven by the viral promoter [102]. It is noteworthy that the miRNA binding sequence with a bulge at positions 9-12 is recommended to avoid the RNA interference-type cleavage and degradation of the sponge RNAs. By comparison, sponges with 4-7 bulged binding sites displayed stronger repressive effects than sponges with two perfect binding sites. Further study demonstrated that lentiviral vectors containing four miR-142 bulged target sequences are more effective at interfering with the natural miRNA targets than those containing four perfectly complementary miRNA binding sequences [106]. In addition, it is worth noting that miRNA sponges showed cross-activity with the miRNA family members [102]. Several research groups adopted the vector-based miRNA-sponge strategy to bind and soak up miRNAs. For example, the miR-31 sponge vectors were introduced in noninvasive MCF-7-RAS cells, inhibiting miR-31 function by > 4.5-fold [107], and a miR-10b-5p sponge vector containing 13 repeats of miR-10b-5p recognition motifs knocked down 75% of miR-10b-5p expression [62]. Such strategies seem to be enthusiastically promising for the functional studies of miRNAs. However, high vector copy number is usually correlated with high risk of immunogenicity or insertional mutagenicity, which limits the clinical usage of the sponge-like constructs. Moreover, complicating the dilemma, the complete set of miRNA targets is, thus far, difficult to determine, which results in the inability to precisely evaluate the vectors’ efficacy. In general, defects in the vectors or expression cassettes should be carefully remedied before the transition towards the clinic.

**Small-molecule drugs**. Apart from the modified oligonucleotide- or expressing vector-based strategies, few research groups studied small-molecule drugs, despite their importance in drugging both the proteome and RNAome (Figure 2B). The pharmaceutical utility of small-molecule compounds in treating human diseases, particularly malignancies, has already achieved great success in the past few years. It is not only the simple reflection of the refinements in small-molecular drug screening methodologies but also in pinpointing the minimal driver oncogenes in cancers, which significantly narrows down the scope of druggable targets [108]. By using the specific miRNA luciferase reporter constructs, azobenzene 2 (the first small-molecule inhibitor of specific miRNAs (SMIRs) that inhibits the pri-miR-21/miR-21-5p expression) [109] and three small-molecule compounds (one activator and two inhibitors that affect miR-122-5p expression) [110] were both screened out from more than 1000 candidate compounds. In those studies, no specific miRNA-small molecule or protein-small molecule interacting mechanism was proposed along with the discoveries of these small molecules, though they were found to alter miRNA expression transcriptionally and specifically. The findings of such small-molecule compounds are no doubt exciting; however the evidence of the specificity supported by using a control reporter of one of nearly two thousand miRNAs is seemingly inconvincible. To some extent, the combination of high-throughput screening and miRNA profiling technologies would possibly solve this problem. In contrast, the small molecule enoxacin rescued globally downregulated miRNAs by binding to TRBP and promoting miRNA processing, representing a novel method to target miRNAs with an established mechanism but a lack of specificity [111]. Due to the defects in the traditional SMIRs screening method, we develop a structure-based virtual screening method to select candidates from The National Cancer Institute (NCI) diversity dataset. We use an MC-fold/MC-sym pipeline to model the 3D structure of pre-miR-21, and select for the potential Dicer binding sites. Then, we conduct the high-throughput docking-based virtual screening using the AutoDock program to choose candidate SMIRs from 1990 NCI diversity compounds. One of the top 5 candidate compounds, AC1MMYR2, is experimentally found to specifically block Dicer processing of pre-miR-21 to mature miR-21-5p. Unexpectedly, the expression of several unrelated miRNAs are also altered. It is of particular interest that AC1MMYR2 inhibits tumorigenesis and invasiveness in mouse orthotopic tumor models and displays tolerated cytotoxicity to host organs, including the liver and kidney [112]. Furthermore, two novel techniques seem very promising for future SMIRs screening. One technique is the design of multimodal small molecules as pre-miRNA ligands (for example aminoglycoside-nucleobase conjugates) [113]. The other one is a lead identification strategy called “Inforna” that integrates advances in RNA structure determination and prediction, identification of RNA motif-small molecule interactions and the scoring of interactions by statistical analyses [114]. Both techniques identify biologically validated SMIRs and show great potential to be refined in concert with the developments of the incorporated techniques. During the past 5 years, we have witnessed an unexpected increase in the development of methods and techniques in small-molecule screening for miRNAs manipulation. Therefore it can be anticipated that the small-molecule drugs for miRNAs can become effective regimens for cancer management in the not-so-distant future. In GBM, AC1MMYR2 has already progressed through the initial step of animal model evaluation. But how far this SMIR goes will be in doubt until the benefits from this drug are proven to be greater than its undesired off-target effects, and it is also the common roadblock that lays ahead of all small molecules on the way to the clinic.

## DELIVERY THROUGH THE BLOOD-BRAIN BARRIER (BBB)

Considerable progress has been made for miRNA manipulation. However, a successful miRNA-based therapeutic must include an effective and safe delivery system, which has become the most challenging barrier for translation into clinical practice. In GBM, the challenge is even greater as the existence of the BBB, a tightly packed layer of endothelial cells surrounding the brain vessels that is a natural barrier designed to shield the brain from exposure to potentially harmful macromolecules in the blood. In the previous proof-of-principle studies, utilization of viral vehicles carrying the expressing cassettes or non-viral vehicles (the most frequently used one is the liposome) encapsulating oligonucleotides has demonstrated effectiveness and tolerated cytotoxicity *in vitro*, triggering great interest for clinical translation. But with respect to the viral delivery system, the enthusiasm is ebbing in treating brain tumors. Because without compromising the BBB integrity, viral carriers are not able to permeate the brain vessels to reach the tumor cells, and prior to their delivery efficiency, the potential for immunostimulation and mutagenesis in humans are more concerning in clinical applications. The demand for a localized intrathecal or intratumoral injection and the safety concerns are formidable for starting a miRNA-targeting trial especially when no precedent has been shown to work. On the contrary, non-viral delivery strategies are capturing the imagination with oligonucleotide and small molecule delivery, triggered by the cumulative interest in intravenous administration of siRNAs and cytotoxic drug formulations. Nanocarriers, which commonly refer to submicron-sized particles ranging from 1 to 1000 nm and carrying therapeutic cargos, are the most studied strategies of the non-viral drug delivery systems. Due to their advantageous properties, including a small size, customizable surface, and improved solubility, the nanoparticles have long been recognized as effective vehicles to penetrate the BBB [115]. In addition to liposomal nanoparticles, currently available nanocarriers can also be fabricated from a variety of synthetic materials, including polymers, amphiphilic star copolymers, dendrimers and inorganics. With advances in material science and chemical engineering, more nano delivery vehicles will be designed in the future to generate the ideal carriers that are expected to be biocompatible, biodegradable, low-toxic, low-immunogenic, and able to bypass rapid hepatic or renal clearance [116]. The flexibility in the conjugation of tumor-targeting and BBB-penetrating ligands has made the nanocarrier a more promising vehicle for drug delivery in the CNS. For example, the porous silica nanocarriers, coated with the cell surface antigen disialoganglioside GD2 antibody, demonstrated tumor-specific delivery of miR-34a-5p in neuroblastoma orthotopic mouse models [117]. More recently, a novel nanocarrier, constructed based on dendrigraft poly-L-lysines (DGL) and polyethyleneglycol (PEG) and conjugated with a cell-penetrating peptide, the nucleolar translocation signal (NoLS) sequence of the LIM Kinase 2 (LIMK2) protein (LIMK2 NoLS peptide, LNP), significantly enhanced in BBB-crossing efficiency and cellular uptake in GBM cells and orthotopic mouse models. Notably, the *in vitro* performance of the BBB-penetrating ability of the DGL-PEG-LNP nanocarriers was better than some other BBB crossing formulations such as lactoferrin-modified nanocarriers and the synthesized compound 10-conjugated nanocarriers [118]. As miRNAs-targeting oligonucleotides and small molecules are becoming promising cargos and the BBB is no long a barrier for the nanocarriers, it is expected that more research will be performed on the development of nanocarrier-delivering miRNA therapeutics in GBM.

## FUTURE PROSPECTS

GBM has become one of the most molecularly characterized malignancies in the past decade. More is understood about the molecular pathogenesis of GBM, boosting the research on targeted agents. However, GBM, thus far, remains a lethal disease with a poor prognosis. The identification of the complex network formed by miRNAs and mRNAs is among the most important discoveries in fundamental research over the past decades, contributing greatly to refine GBM research. Thereafter the understanding of initiation, progression, and dissemination of GBM is no long limited to the protein-coding genome. The small non-coding RNA transcripts are not nonsense at all but instead ubiquitously participate in the hallmark features of cancer. Therefore, the utilization of miRNAs as therapeutic targets is conceptually accepted as an alternative approach in molecular oncology ranslational research. In some diseases, miRNA-targeting therapeutics have been used in clinical trials, such as Miravirsen in hepatitis C virus infection (LNA-antimiR-122-5p, phase IIa, NCT01200420), MRX34 in primary liver cancer or metastatic cancer (miR-34a-5p mimics, phase I, NCT01829971) and Targomir in malignant pleural mesothelioma (miR-15a-5p/16-5p mimics, phase I, NCT02369198) (https://www.clinicaltrials.gov). In GBM, clinical translation is anticipated because many functional miRNAs have already been available as therapeutic targets. For example, the restoration of miR-34a-5p, a tumor suppressive miRNA that simultaneously downregulates the expressions of MET, PDGFRA and CDK6, can possibly outperform any single targeted agents tailored to those targets [37, 39]. Likewise, the depletion of miR-10b-5p can replenish multiple tumor suppressors, including TP53 and p16^INK4a^, jointly repressing GBM growth [62, 63]. Moreover, the nature of miRNAs, by which they simultaneously occur at the regulation of multiple genes from the same or different pathways, theoretically reduces the possibility for drug resistance in GBM in response to monotherapies. Though, thus far, no clinical trial on miRNA intervention has been conducted in GBM, we can still enthusiastically envision a bright future for miRNA-targeting therapeutics as therapeutic cargos and BBB-penetrating delivery systems are already available.

## SUPPLEMENTARY MATERIAL



## References

[1] Wen PY, Kesari S (2008). Malignant gliomas in adults. N Engl J Med.

[2] Stupp R, Hegi ME, Mason WP, van den Bent MJ, Taphoorn MJB, Janzer RC, Ludwin SK, Allgeier A, Fisher B, Belanger K, Hau P, Brandes AA, Gijtenbeek J (2009). Effects of radiotherapy with concomitant and adjuvant temozolomide versus radiotherapy alone on survival in glioblastoma in a randomised phase III study: 5-year analysis of the EORTC-NCIC trial. Lancet Oncol.

[3] Ruby JG, Jan CH, Bartel DP (2007). Intronic microRNA precursors that bypass Drosha processing. Nature.

[4] Kim VN, Han J, Siomi MC (2009). Biogenesis of small RNAs in animals. Nat Rev Mol Cell Biol.

[5] Pasquinelli AE (2012). MicroRNAs and their targets: recognition, regulation and an emerging reciprocal relationship. Nat Rev Genet.

[6] Huntzinger E, Izaurralde E (2011). Gene silencing by microRNAs: contributions of translational repression and mRNA decay. Nat Rev Genet.

[7] Friedman RC, Farh KK-H, Burge CB, Bartel DP (2009). Most mammalian mRNAs are conserved targets of microRNAs. Genome Res.

[8] Salmena L, Poliseno L, Tay Y, Kats L, Pandolfi PP (2011). A ceRNA hypothesis: the Rosetta Stone of a hidden RNA language?. Cell.

[9] Sumazin P, Yang X, Chiu H-S, Chung W-J, Iyer A, Llobet-Navas D, Rajbhandari P, Bansal M, Guarnieri P, Silva J, Califano A (2011). An Extensive MicroRNA-Mediated Network of RNA-RNA Interactions Regulates Established Oncogenic Pathways in Glioblastoma. Cell.

[10] Tay Y, Kats L, Salmena L, Weiss D, Tan SM, Ala U, Karreth F, Poliseno L, Provero P, Di Cunto F, Lieberman J, Rigoutsos I, Pandolfi PP (2011). Coding-independent regulation of the tumor suppressor PTEN by competing endogenous mRNAs. Cell.

[11] Yoon J-H, Abdelmohsen K, Srikantan S, Yang X, Martindale JL, De S, Huarte M, Zhan M, Becker KG, Gorospe M (2012). LincRNA-p21 suppresses target mRNA translation. Molecular Cell.

[12] Chiyomaru T, Yamamura S, Fukuhara S, Yoshino H, Kinoshita T, Majid S, Saini S, Chang I, Tanaka Y, Enokida H, Seki N, Nakagawa M, Dahiya R (2013). Genistein inhibits prostate cancer cell growth by targeting miR-34a and oncogenic HOTAIR. PLoS ONE.

[13] Tsang FHC, Au SLK, Wei L, Fan DNY, Lee JMF, Wong CCL, Ng IOL, Wong C-M (2015). Long non-coding RNA HOTTIP is frequently up-regulated in hepatocellular carcinoma and is targeted by tumour suppressive miR-125b. Liver Int.

[14] Calin GA, Liu C-G, Ferracin M, Hyslop T, Spizzo R, Sevignani C, Fabbri M, Cimmino A, Lee EJ, Wojcik SE, Shimizu M, Tili E, Rossi S (2007). Ultraconserved regions encoding ncRNAs are altered in human leukemias and carcinomas. Cancer Cell.

[15] Poliseno L, Salmena L, Zhang J, Carver B, Haveman WJ, Pandolfi PP (2010). A coding-independent function of gene and pseudogene mRNAs regulates tumour biology. Nature.

[16] Rutnam ZJ, Du WW, Yang W, Yang X, Yang BB (2014). The pseudogene TUSC2P promotes TUSC2 function by binding multiple microRNAs. Nat Commun.

[17] Esposito F, De Martino M, Petti MG, Forzati F, Tornincasa M, Federico A, Arra C, Pierantoni GM, Fusco A (2014). HMGA1 pseudogenes as candidate proto-oncogenic competitive endogenous RNAs. Oncotarget.

[18] Memczak S, Jens M, Elefsinioti A, Torti F, Krueger J, Rybak A, Maier L, Mackowiak SD, Gregersen LH, Munschauer M, Loewer A, Ziebold U, Landthaler M (2013). Circular RNAs are a large class of animal RNAs with regulatory potency. Nature.

[19] Hansen TB, Jensen TI, Clausen BH, Bramsen JB, Finsen B, Damgaard CK, Kjems J (2013). Natural RNA circles function as efficient microRNA sponges. Nature.

[20] Okamura K, Phillips MD, Tyler DM, Duan H, Chou Y-T, Lai EC (2008). The regulatory activity of microRNA* species has substantial influence on microRNA and 3′ UTR evolution. Nat Struct Mol Biol.

[21] Kozomara A, Griffiths-Jones S (2014). miRBase: annotating high confidence microRNAs using deep sequencing data. Nucleic Acids Res.

[22] Chan JA, Krichevsky AM, Kosik KS (2005). MicroRNA-21 is an antiapoptotic factor in human glioblastoma cells. Cancer Res.

[23] Ciafrè SA, Galardi S, Mangiola A, Ferracin M, Liu CG, Sabatino G, Negrini M, Maira G, Croce CM, Farace MG (2005). Extensive modulation of a set of microRNAs in primary glioblastoma. Biochem Biophys Res Commun.

[24] Furnari FB, Fenton T, Bachoo RM, Mukasa A, Stommel JM, Stegh A, Hahn WC, Ligon KL, Louis DN, Brennan C, Chin L, DePinho RA, Cavenee WK (2007). Malignant astrocytic glioma: genetics, biology, and paths to treatment. Genes Dev.

[25] Hanahan D, Weinberg RA (2011). Hallmarks of Cancer: The Next Generation. Cell.

[26] Weinstein JN, Collisson EA, Mills GB, Shaw KRM, Ozenberger BA, Ellrott K, Shmulevich I, Sander C, Stuart JM, Cancer Genome Atlas Research Network (2013). The Cancer Genome Atlas Pan-Cancer analysis project. Nat Genet.

[27] Brennan CW, Verhaak RGW, McKenna A, Campos B, Noushmehr H, Salama SR, Zheng S, Chakravarty D, Sanborn JZ, Berman SH, Beroukhim R, Bernard B, Wu C-J (2013). The Somatic Genomic Landscape of Glioblastoma. Cell.

[28] Lu J, Getz G, Miska EA, Alvarez-Saavedra E, Lamb J, Peck D, Sweet-Cordero A, Ebert BL, Mak RH, Ferrando AA, Downing JR, Jacks T, Horvitz HR (2005). MicroRNA expression profiles classify human cancers. Nature.

[29] Li Y, Xu J, Chen H, Bai J, Li S, Zhao Z, Shao T, Jiang T, Ren H, Kang C, Li X (2013). Comprehensive analysis of the functional microRNA-mRNA regulatory network identifies miRNA signatures associated with glioma malignant progression. Nucleic Acids Res.

[30] Kim TM, Huang W, Park R, Park PJ, Johnson MD (2011). A Developmental Taxonomy of Glioblastoma Defined and Maintained by MicroRNAs. Cancer Res.

[31] Lemmon MA, Schlessinger J (2010). Cell signaling by receptor tyrosine kinases. Cell.

[32] McLendon R, Friedman A, Bigner D, Van Meir EG, Brat DJ, M Mastrogianakis G, Olson JJ, Mikkelsen T, Lehman N, Aldape K, Alfred Yung WK, Bogler O, VandenBerg S (2008). Comprehensive genomic characterization defines human glioblastoma genes and core pathways. Nature.

[33] Kefas B, Godlewski J, Comeau L, Li Y, Abounader R, Hawkinson M, Lee J, Fine H, Chiocca EA, Lawler S, Purow B (2008). microRNA-7 inhibits the epidermal growth factor receptor and the Akt pathway and is down-regulated in glioblastoma. Cancer Res.

[34] Papagiannakopoulos T, Friedmann-Morvinski D, Neveu P, Dugas JC, Gill RM, Huillard E, Liu C, Zong H, Rowitch DH, Barres BA, Verma IM, Kosik KS (2012). Pro-neural miR-128 is a glioma tumor suppressor that targets mitogenic kinases. Oncogene.

[35] Li X, Liu Y, Granberg KJ, Wang Q, Moore LM, Ji P, Gumin J, Sulman EP, Calin GA, Haapasalo H, Nykter M, Shmulevich I, Fuller GN (2015). Two mature products of MIR-491 coordinate to suppress key cancer hallmarks in glioblastoma. Oncogene.

[36] Mathew LK, Skuli N, Mucaj V, Lee SS, Zinn PO, Sathyan P, Imtiyaz HZ, Zhang Z, Davuluri RV, Rao S, Venneti S, Lal P, Lathia JD (2014). miR-218 opposes a critical RTK-HIF pathway in mesenchymal glioblastoma. Proc Natl Acad Sci U S A.

[37] Genovese G, Ergun A, Shukla SA, Campos B, Hanna J, Ghosh P, Quayle SN, Rai K, Colla S, Ying H, Wu C-J, Sarkar S, Xiao Y (2012). microRNA regulatory network inference identifies miR-34a as a novel regulator of TGF-β signaling in glioblastoma. Cancer Discov.

[38] Verhaak RGW, Hoadley KA, Purdom E, Wang V, Qi Y, Wilkerson MD, Miller CR, Ding L, Golub T, Mesirov JP, Alexe G, Lawrence M, O'Kelly M (2010). Integrated genomic analysis identifies clinically relevant subtypes of glioblastoma characterized by abnormalities in PDGFRA, IDH1, EGFR, and NF1. Cancer Cell.

[39] Li Y, Guessous F, Zhang Y, Dipierro C, Kefas B, Johnson E, Marcinkiewicz L, Jiang J, Yang Y, Schmittgen TD, Lopes B, Schiff D, Purow B (2009). MicroRNA-34a inhibits glioblastoma growth by targeting multiple oncogenes. Cancer Res.

[40] Liu P, Cheng H, Roberts TM, Zhao JJ (2009). Targeting the phosphoinositide 3-kinase pathway in cancer. Nat Rev Drug Discov.

[41] Karnoub AE, Weinberg RA (2008). Ras oncogenes: split personalities. Nat Rev Mol Cell Biol.

[42] Lo H-W (2010). Targeting Ras-RAF-ERK and its interactive pathways as a novel therapy for malignant gliomas. Curr Cancer Drug Targets.

[43] Wang L, Shi Z-M, Jiang C-F, Liu X, Chen Q-D, Qian X, Li D-M, Ge X, Wang X-F, Liu L-Z, You Y-P, Liu N, Jiang B-H (2014). MiR-143 acts as a tumor suppressor by targeting N-RAS and enhances temozolomide-induced apoptosis in glioma. Oncotarget.

[44] Wang X-R, Luo H, Li H-L, Cao L, Wang X-F, Yan W, Wang Y-Y, Zhang J-X, Jiang T, Kang C-S, Liu N, You Y-P, Chinese Glioma Cooperative Group (CGCG) (2013). Overexpressed let-7a inhibits glioma cell malignancy by directly targeting K-ras, independently of PTEN. Neuro Oncol.

[45] Shi Z, Chen Q, Li C, Wang L, Qian X, Jiang C, Liu X, Wang X, Li H, Kang C, Jiang T, Liu LZ, You Y (2014). MiR-124 governs glioma growth and angiogenesis and enhances chemosensitivity by targeting R-Ras and N-Ras. Neuro Oncol.

[46] Tan X, Wang S, Yang B, Zhu L, Yin B, Chao T, Zhao J, Yuan J, Qiang B, Peng X (2012). The CREB-miR-9 negative feedback minicircuitry coordinates the migration and proliferation of glioma cells. PLoS ONE.

[47] Malzkorn B, Wolter M, Liesenberg F, Grzendowski M, Stühler K, Meyer HE, Reifenberger G (2010). Identification and functional characterization of microRNAs involved in the malignant progression of gliomas. Brain Pathol.

[48] Stambolic V, Suzuki A, La Pompa De JL, Brothers GM, Mirtsos C, Sasaki T, Ruland J, Penninger JM, Siderovski DP, Mak TW (1998). Negative regulation of PKB/Akt-dependent cell survival by the tumor suppressor PTEN. Cell.

[49] Huse JT, Brennan C, Hambardzumyan D, Wee B, Pena J, Rouhanifard SH, Sohn-Lee C, le Sage C, Agami R, Tuschl T, Holland EC (2009). The PTEN-regulating microRNA miR-26a is amplified in high-grade glioma and facilitates gliomagenesis *in vivo*. Genes Dev.

[50] Li H, Yang BB (2012). Stress response of glioblastoma cells mediated by miR-17-5p targeting PTEN and the passenger strand miR-17-3p targeting MDM2. Oncotarget.

[51] Tan X, Wang S, Zhu L, Wu C, Yin B, Zhao J, Yuan J, Qiang B, Peng X (2012). cAMP response element-binding protein promotes gliomagenesis by modulating the expression of oncogenic microRNA-23a. Proc Natl Acad Sci U S A.

[52] Kleihues P, Burger PC, Scheithauer BW (1993). The new WHO classification of brain tumours. Brain Pathol.

[53] Plate KH, Risau W (1995). Angiogenesis in malignant gliomas. Glia.

[54] Yue X, Wang P, Xu J, Zhu Y, Sun G, Pang Q, Tao R (2012). MicroRNA-205 functions as a tumor suppressor in human glioblastoma cells by targeting VEGF-A. Oncol Rep.

[55] Shweiki D, Itin A, Soffer D, Keshet E (1992). Vascular endothelial growth factor induced by hypoxia may mediate hypoxia-initiated angiogenesis. Nature.

[56] Harris AL (2002). Hypoxia—a key regulatory factor in tumour growth. Nat Rev Cancer.

[57] Chen L, Han L, Zhang K, Shi Z, Zhang J, Zhang A, Wang Y, Song Y, Li Y, Jiang T, Pu P, Jiang C, Kang C (2012). VHL regulates the effects of miR-23b on glioma survival and invasion via suppression of HIF-1α/VEGF and β-catenin/Tcf-4 signaling. Neuro Oncol.

[58] Zhang K-L, Han L, Chen L-Y, Shi Z-D, Yang M, Ren Y, Chen L-C, Zhang J-X, Pu P-Y, Kang C-S (2014). Blockage of a miR-21/EGFR regulatory feedback loop augments anti-EGFR therapy in glioblastomas. Cancer Lett.

[59] Babae N, Bourajjaj M, Liu Y, Van Beijnum JR, Cerisoli F, Scaria PV, Verheul M, Van Berkel MP, Pieters EHE, Van Haastert RJ, Yousefi A, Mastrobattista E, Storm G (2014). Systemic miRNA-7 delivery inhibits tumor angiogenesis and growth in murine xenograft glioblastoma. Oncotarget.

[60] Arrillaga-Romany I, Reardon DA, Wen PY (2014). Current status of antiangiogenic therapies for glioblastomas. Expert Opin Investig Drugs.

[61] Kruiswijk F, Labuschagne CF, Vousden KH (2015). p53 in survival, death and metabolic health: a lifeguard with a licence to kill. Nat Rev Mol Cell Biol.

[62] Lin J, Teo S, Lam DH, Jeyaseelan K, Wang S (2012). MicroRNA-10b pleiotropically regulates invasion, angiogenicity and apoptosis of tumor cells resembling mesenchymal subtype of glioblastoma multiforme. Cell Death Dis.

[63] Gabriely G, Yi M, Narayan RS, Niers JM, Würdinger T, Imitola J, Ligon KL, Kesari S, Esau C, Stephens RM, Tannous BA, Krichevsky AM (2011). Human glioma growth is controlled by microRNA-10b. Cancer Res.

[64] Brown CJ, Lain S, Verma CS, Fersht AR, Lane DP (2009). Awakening guardian angels: drugging the p53 pathway. Nat Rev Cancer.

[65] Suh S-S, Yoo JY, Nuovo GJ, Jeon Y-J, Kim S, Lee TJ, Kim T, Bakàcs A, Alder H, Kaur B, Aqeilan RI, Pichiorri F, Croce CM (2012). MicroRNAs/TP53 feedback circuitry in glioblastoma multiforme. Proc Natl Acad Sci U S A.

[66] Sun Y-C, Wang J, Guo C-C, Sai K, Wang J, Chen F-R, Yang Q-Y, Chen Y-S, Wang J, To TS-S, Zhang Z-P, Mu Y-G, Chen Z-P (2014). MiR-181b sensitizes glioma cells to teniposide by targeting MDM2. BMC Cancer.

[67] Skalsky RL, Cullen BR (2011). Reduced expression of brain-enriched microRNAs in glioblastomas permits targeted regulation of a cell death gene. PLoS ONE.

[68] Burkhart DL, Sage J (2008). Cellular mechanisms of tumour suppression by the retinoblastoma gene. Nat Rev Cancer.

[69] Kim H, Huang W, Jiang X, Pennicooke B, Park PJ, Johnson MD (2010). Integrative genome analysis reveals an oncomir/oncogene cluster regulating glioblastoma survivorship. Proc Natl Acad Sci U S A.

[70] Lundberg AS, Weinberg RA (1998). Functional inactivation of the retinoblastoma protein requires sequential modification by at least two distinct cyclin-cdk complexes. Mol Cell Biol.

[71] Deng X, Ma L, Wu M, Zhang G, Jin C, Guo Y, Liu R (2013). miR-124 radiosensitizes human glioma cells by targeting CDK4. J Neurooncol.

[72] Qiu S, Huang D, Yin D, Li F, Li X, Kung H-F, Peng Y (2013). Suppression of tumorigenicity by microRNA-138 through inhibition of EZH2-CDK4/6-pRb-E2F1 signal loop in glioblastoma multiforme. Biochim Biophys Acta.

[73] Hui W, Yuntao L, Lun L, WenSheng L, ChaoFeng L, HaiYong H, Yueyang B (2013). MicroRNA-195 inhibits the proliferation of human glioma cells by directly targeting cyclin D1 and cyclin E1. PLoS ONE.

[74] Serrano M, Hannon GJ, Beach D (1993). A new regulatory motif in cell-cycle control causing specific inhibition of cyclin D/CDK4. Nature.

[75] Hamilton JD, Rapp M, Schneiderhan T, Marcel Schneiderhan T, Sabel M, Hayman A, Scherer A, Kröpil P, Budach W, Gerber P, Kretschmar U, Arne Gerber P, Prabhu S (2014). Glioblastoma multiforme metastasis outside the CNS: three case reports and possible mechanisms of escape. J Clin Oncol.

[76] Cuddapah VA, Robel S, Watkins S, Sontheimer H (2014). A neurocentric perspective on glioma invasion. Nat Rev Neurosci.

[77] Vandenbroucke RE, Libert C (2014). Is there new hope for therapeutic matrix metalloproteinase inhibition?. Nat Rev Drug Discov.

[78] Yan W, Zhang W, Sun L, Liu Y, You G, Wang Y, Kang C, You Y, Jiang T (2011). Identification of MMP-9 specific microRNA expression profile as potential targets of anti-invasion therapy in glioblastoma multiforme. Brain Res.

[79] Asuthkar S, Velpula KK, Chetty C, Gorantla B, Rao JS (2012). Epigenetic regulation of miRNA-211 by MMP-9 governs glioma cell apoptosis, chemosensitivity and radiosensitivity. Oncotarget.

[80] Zheng X, Chopp M, Lu Y, Buller B, Jiang F (2013). MiR-15b and miR-152 reduce glioma cell invasion and angiogenesis via NRP-2 and MMP-3. Cancer Lett.

[81] Lu Y, Chopp M, Zheng X, Katakowski M, Buller B, Jiang F (2013). MiR-145 reduces ADAM17 expression and inhibits *in vitro* migration and invasion of glioma cells. Oncol Rep.

[82] Gabriely G, Würdinger T, Kesari S, Esau CC, Burchard J, Linsley PS, Krichevsky AM (2008). MicroRNA 21 promotes glioma invasion by targeting matrix metalloproteinase regulators. Mol Cell Biol.

[83] Hood JD, Cheresh DA (2002). Role of integrins in cell invasion and migration. Nat Rev Cancer.

[84] Irradiation differentially affects substratum-dependent survival, adhesion, and invasion of glioblastoma cell lines (2003).

[85] Fowler A, Thomson D, Giles K, Maleki S, Mreich E, Wheeler H, Leedman P, Biggs M, Cook R, Little N, Robinson B, McDonald K (2011). miR-124a is frequently down-regulated in glioblastoma and is involved in migration and invasion. Eur J Cancer.

[86] Boutla A, Delidakis C, Tabler M (2003). Developmental defects by antisense-mediated inactivation of micro-RNAs 2 and 13 in Drosophila and the identification of putative target genes. Nucleic Acids Res.

[87] Hutvágner G, Simard MJ, Mello CC, Zamore PD (2004). Sequence-specific inhibition of small RNA function. PLoS Biol.

[88] Meister G, Landthaler M, Dorsett Y, Tuschl T (2004). Sequence-specific inhibition of microRNA- and siRNA-induced RNA silencing. RNA.

[89] Esau C, Davis S, Murray SF, Yu XX, Pandey SK, Pear M, Watts L, Booten SL, Graham M, McKay R, Subramaniam A, Propp S, Lollo BA, Monia BP (2006). miR-122 regulation of lipid metabolism revealed by *in vivo* antisense targeting. Cell Metab.

[90] Davis S, Lollo B, Freier S, Esau C (2006). Improved targeting of miRNA with antisense oligonucleotides. Nucleic Acids Res.

[91] Petersen M, Wengel J (2003). LNA: a versatile tool for therapeutics and genomics. Trends Biotechnol.

[92] Obad S, Santos dos CO, Petri A, Heidenblad M, Broom O, Ruse C, Fu C, Lindow M, Stenvang J, Straarup EM, Hansen HF, Koch T, Pappin D (2011). Silencing of microRNA families by seed-targeting tiny LNAs. Nat Genet.

[93] Cummins LL, Owens SR, Risen LM, Lesnik EA, Freier SM, McGee D, Guinosso CJ, Cook PD (1995). Characterization of fully 2′-modified oligoribonucleotide hetero- and homoduplex hybridization and nuclease sensitivity. Nucleic Acids Res.

[94] Krützfeldt J, Rajewsky N, Braich R, Rajeev KG, Tuschl T, Manoharan M, Stoffel M (2005). Silencing of microRNAs *in vivo* with “antagomirs”. Nature.

[95] Wolfrum C, Shi S, Jayaprakash KN, Jayaraman M, Wang G, Pandey RK, Rajeev KG, Nakayama T, Charrise K, Ndungo EM, Zimmermann T, Koteliansky V, Manoharan M, Stoffel M (2007). Mechanisms and optimization of *in vivo* delivery of lipophilic siRNAs. Nat Biotechnol.

[96] Thum T, Gross C, Fiedler J, Fischer T, Kissler S, Bussen M, Galuppo P, Just S, Rottbauer W, Frantz S, Castoldi M, Soutschek J, Koteliansky V (2008). MicroRNA-21 contributes to myocardial disease by stimulating MAP kinase signalling in fibroblasts. Nature.

[97] Ma L, Reinhardt F, Pan E, Soutschek J, Bhat B, Marcusson EG, Teruya-Feldstein J, Bell GW, Weinberg RA (2010). Therapeutic silencing of miR-10b inhibits metastasis in a mouse mammary tumor model. Nat Biotechnol.

[98] Robb GB, Brown KM, Khurana J, Rana TM (2005). Specific and potent RNAi in the nucleus of human cells. Nat Struct Mol Biol.

[99] Birmingham A, Anderson E, Sullivan K, Reynolds A, Boese Q, Leake D, Karpilow J, Khvorova A (2007). A protocol for designing siRNAs with high functionality and specificity. Nat Protoc.

[100] Wiggins JF, Ruffino L, Kelnar K, Omotola M, Patrawala L, Brown D, Bader AG (2010). Development of a lung cancer therapeutic based on the tumor suppressor microRNA-34. Cancer Res.

[101] Guennewig B, Roos M, Dogar AM, Gebert LFR, Zagalak JA, Vongrad V, Metzner KJ, Hall J (2014). Synthetic pre-microRNAs reveal dual-strand activity of miR-34a on TNF-α. RNA.

[102] Ebert MS, Neilson JR, Sharp PA (2007). MicroRNA sponges: competitive inhibitors of small RNAs in mammalian cells. Nat Meth.

[103] Carè A, Catalucci D, Felicetti F, Bonci D, Addario A, Gallo P, Bang M-L, Segnalini P, Gu Y, Dalton ND, Elia L, Latronico MVG, Høydal M (2007). MicroRNA-133 controls cardiac hypertrophy. Nat Med.

[104] Sayed D, Rane S, Lypowy J, He M, Chen I-Y, Vashistha H, Yan L, Malhotra A, Vatner D, Abdellatif M (2008). MicroRNA-21 targets Sprouty2 and promotes cellular outgrowths. Mol Biol Cell.

[105] Scherr M, Venturini L, Battmer K, Schaller-Schoenitz M, Schaefer D, Dallmann I, Ganser A, Eder M (2007). Lentivirus-mediated antagomir expression for specific inhibition of miRNA function. Nucleic Acids Res.

[106] Gentner B, Schira G, Giustacchini A, Amendola M, Brown BD, Ponzoni M, Naldini L (2008). Stable knockdown of microRNA *in vivo* by lentiviral vectors. Nat Meth.

[107] Valastyan S, Reinhardt F, Benaich N, Calogrias D, SzAsz AM, Wang ZC, Brock JE, Richardson AL, Weinberg RA (2009). A Pleiotropically Acting MicroRNA, miR-31, Inhibits Breast Cancer Metastasis. Cell.

[108] Crews CM (2010). Targeting the Undruggable Proteome: The Small Molecules of My Dreams. Chem Biol.

[109] Gumireddy K, Young DD, Xiong X, Hogenesch JB, Huang Q, Deiters A (2008). Small-molecule inhibitors of microrna miR-21 function. Angew Chem Int Ed Engl.

[110] Young DD, Connelly CM, Grohmann C, Deiters A (2010). Small molecule modifiers of microRNA miR-122 function for the treatment of hepatitis C virus infection and hepatocellular carcinoma. J Am Chem Soc.

[111] Melo S, Villanueva A, Moutinho C, Davalos V, Spizzo R, Ivan C, Rossi S, Setien F, Casanovas O, Simo-Riudalbas L, Carmona J, Carrere J, Vidal A, Aytes A (2011). Small molecule enoxacin is a cancer-specific growth inhibitor that acts by enhancing TAR RNA-binding protein 2-mediated microRNA processing. Proc Natl Acad Sci U S A.

[112] Shi Z, Zhang J, Qian X, Han L, Zhang K, Chen L, Liu J, Ren Y, Yang M, Zhang A, Pu P, Kang C (2013). AC1MMYR2, an inhibitor of dicer-mediated biogenesis of Oncomir miR-21, reverses epithelial-mesenchymal transition and suppresses tumor growth and progression. Cancer Res.

[113] Vo DD, Staedel C, Zehnacker L, Benhida R, Darfeuille F, Duca M (2014). Targeting the production of oncogenic microRNAs with multimodal synthetic small molecules. ACS Chem Biol.

[114] Velagapudi SP, Gallo SM, Disney MD (2014). Sequence-based design of bioactive small molecules that target precursor microRNAs. Nat Chem Biol.

[115] Gomes MJ, Martins S, Sarmento B (2015). siRNA as a tool to improve the treatment of brain diseases: Mechanism, targets and delivery. Ageing Res Rev.

[116] Wang H, Jiang Y, Peng H, Chen Y, Zhu P, Huang Y (2015). Recent progress in microRNA delivery for cancer therapy by non-viral synthetic vectors. Adv Drug Deliv Rev.

[117] Tivnan A, Orr WS, Gubala V, Nooney R, Williams DE, McDonagh C, Prenter S, Harvey H, Domingo-Fernández R, Bray IM, Piskareva O, Ng CY, Lode HN (2012). Inhibition of neuroblastoma tumor growth by targeted delivery of microRNA-34a using anti-disialoganglioside GD2 coated nanoparticles. PLoS ONE.

[118] Yao H, Wang K, Wang Y, Wang S, Li J, Lou J, Ye L, Yan X, Lu W, Huang R (2015). Enhanced blood-brain barrier penetration and glioma therapy mediated by a new peptide modified gene delivery system. Biomaterials.

